# Dynamin-Related Protein 1 Is Involved in Mitochondrial Damage, Defective Mitophagy, and NLRP3 Inflammasome Activation Induced by MSU Crystals

**DOI:** 10.1155/2022/5064494

**Published:** 2022-10-25

**Authors:** Hui Jiang, Feng Chen, DianZe Song, Xiaoqin Zhou, Long Ren, Mei Zeng

**Affiliations:** ^1^Institute of Basic Medicine and Forensic Medicine, North Sichuan Medical College and Institute of Rheumatology and Immunology, The Affiliated Hospital of North Sichuan Medical College, No. 1 South Maoyuan Road, Nanchong, 637001 Sichuan, China; ^2^Medical Imaging Key Laboratory of Sichuan Province, The Affiliated Hospital of North Sichuan Medical College, No. 1 South Maoyuan Road, Nanchong, 637001 Sichuan, China; ^3^The Fifth People's Hospital of Nanchong City, 21# Bajiao Street, Nanchong, 637100 Sichuan, China

## Abstract

Excessive generation of reactive oxygen species (ROS) has great impacts on MSU crystal-induced inflammation. Drp1-dependent mitochondrial fission is closely associated with mitochondrial ROS levels. However, whether Drp1 signaling contributes to MSU crystal-induced inflammation remains unclear. Mice bone marrow-derived macrophages (BMDMs) were primed with LPS and then stimulated with MSU suspensions for 12 h. The protein levels associated with mitochondrial dynamics, oxidative stress, and mitophagy were detected by Western blot. BMDMs were loaded with MitoTracker Green probe to detect mitochondrial morphology. To measure mitochondrial reactive oxygen species (ROS) and total ROS levels, cells were loaded, respectively, with MitoSOX and DHE probes. The effects of Mito-TEMPO, an antioxidant that targets the mitochondria or DRP1 inhibitor (Mdivi-1) on MSU crystal-induced peritonitis and arthritis mouse models, were evaluated. Our study revealed that MSU crystal stimulation resulted in elevation of mitochondrial fragmentation of BMDMs. Treatment with Mito-TEMPO or Drp1 knockdown significantly ameliorated the mitochondrial damage induced by MSU crystals. BMDMs exposure to MSU crystals increased the expression of auto/mitophagy marker proteins and promoted the fusion of mitophagosomes with lysosomes, leading to accumulation of mitolysosomes. Drp1 knockdown alleviated defective mitophagy and activation of the NLRP3 inflammasome in MSU crystal-treated BMDMs. This study indicates that there is crosstalk between mitochondrial ROS and Drp1 signaling in MSU crystal-induced inflammation. Drp1 signaling is involved in MSU crystal-induced mitochondrial damage, impaired mitophagy and NLRP3 inflammasome activation.

## 1. Introduction

Gout is the most common form of inflammatory arthritis worldwide, and it is caused by the deposition of monosodium urate (MSU) crystals in joints and surrounding tissues [1]. In high-income countries, the prevalence of gout appears to have stabilized [2, 3]. However, the incidence of gout is still increasing in mainland China [4]. The main treatment options for gout flare up are colchicine, nonsteroidal anti-inflammatory drugs (NSAIDs), or corticosteroids [5–7]. However, these traditional treatments have many side effects, and it is still difficult to treat gout [8]. IL-1 inhibitors are commonly used in patients with intolerable side effects or contraindications to first-line anti-inflammatory therapies, but patients must be closely monitored for adverse reactions or intolerance.

MSU crystals stimulate innate immune pathways by damage-associated molecular patterns. In addition, studies have confirmed that excessive ROS production plays a key role in mediating inflammation in gout patients [9, 10]. However, the exact molecular mechanism by which ROS regulate the inflammatory response induced by MSU crystals remains unknown. Mitochondria are the main sources of ROS, and damaged mitochondria produce higher ROS levels. Mitochondrial networks are dynamic and respond to metabolic signals by increasing the mass of the network (biogenesis) as well as by dividing existing mitochondria to form a larger network (fission) [11]. Mitochondrial biogenesis increases mitochondrial mass, and mitochondrial fission increases the actual number of mitochondria.

Dynamin-related protein 1 (Drp1) plays an important role during the process of mitochondrial fission [12–15]. Phosphorylated and activated Drp1 is recruited from the cytoplasm to a division site near the outer mitochondrial membrane, subsequently initiating fission. An increasing number of studies have shown that Drp1 activation leads to an increase in mitochondrial fission, thereby accelerating ROS production, while Drp1 inhibition leads to decreased ROS levels [16]. Drp1-mediated fission is considered to be the convergence point of ROS-dependent pathological processes. ROS may be upstream molecules of Drp1 activation, resulting in mitochondrial fission [13]. Under conditions of excessive ROS levels, enhanced mitochondrial fragmentation can also be observed, and this increased mitochondrial fragmentation is accompanied by increased Drp1 activity [14]. Therefore, the impact of Drp1 on ROS production appears to be bidirectional: on the one hand, Drp1may act as a receiver of ROS signals, and on the other hand, it stimulates ROS production by regulating mitochondrial fission.

Excessive generation of mitochondrial ROS can cause mitochondrial dysfunction. To protect normal cellular function, damaged mitochondria is eliminated by mitophagy [17]. PINK1 (PTEN-induced putative kinase 1)-PRKN/Parkin (parkin RBR E3 ubiquitin protein ligase)-OPTN (optineurin) proteins sense damaged mitochondria; then, this sensing triggers and recruits autophagy-related proteins (such as BECN1/Beclin1, MAP1LC3B/LC3B, and SQSTM1/P62) to damaged mitochondria [18], eventually resulting in the formation of mitophagosomes. Finally, mitophagosomes containing damaged mitochondria are transported to lysosomes for clearance. Multiple signaling pathways are involved in mitophagy, by which damaged mitochondria is eliminated from cells by fusion with lysosomes. However, blockage of mitophagy leads to the accumulation of damaged mitochondria and exacerbates inflammation [19–21].

Previous studies have suggested that mitochondrial fission occurs first, and then mitophagosomes are formed [22]. Based on these reports, we summarized the relationship among mitochondrial damage, mitochondrial fission, and mitophagy caused by oxidative stress (Supplementary Figure 1). However, the link between mitochondrial fission and mitophagy in MSU crystal-induced inflammation is poorly understood. The underlying molecular mechanism by which MSU crystals affect mitophagy is also unclear. Based on these facts, in the present study, we explored whether there was crosstalk between mitochondrial ROS and Drp1 signaling in response to exposure to MSU crystals. The roles of Drp1 signaling in MSU crystal-induced mitochondrial damage, mitophagy, and NLRP3 inflammasome activation were also assessed. Our data showed that Drp1 knockdown could improve MSU crystal-induced inflammatory response by inhibiting mitochondrial damage, impaired mitophagy, and NLRP3 inflammasome activation.

## 2. Materials and Methods

### 2.1. Mice

C57BL/6 mice aged 8 to 10 weeks were ordered from the Dossy Experimental Animals Company (Chengdu, China). All the animal experiments were performed in accordance with the Guidelines for the Care and Use of Experimental Animals and were approved by the Ethics Committee of North Sichuan Medical College.

### 2.2. Cell Culture, Stimulation, and Transfection

Bone marrow-derived macrophages (BMDMs) were isolated from C57BL/6 mice aged 8-10 weeks as described [23] The macrophages were cultured in high-glucose DMEM supplemented with 10% fetal bovine serum (FBS), M-CSF (50 ng/ml), and 100 U/ml penicillin-streptomycin for 5-7 days. For Mito-TEMPO treatment, Mito-TEMPO (20 *μ*M, 14 h)-treated BMDMs were primed with LPS (100 ng/ml) for 1 h and then stimulated with MSU crystals for 12 h. When BMDMs were treated with MSU suspensions, and cultured in Opti-MEM Reduced Serum Medium (Gibco), the siRNA targeting Drp1, PINK1, or BECN1 were synthesized by Sangon Biotechnology, and their sequences are shown in supplementary Table 1. The plasmids were ordered from Jingmai Biotechnology (Nanjing, China). The siRNA and plasmid transfection were used, respectively, LipoRNAi and Lipo8000 kit (Beyotime, China), and followed the manufacturer's instructions.

### 2.3. Immunofluorescence and Confocal Microscopy

BMDMs were fixed in 4% paraformaldehyde (PFA), permeabilized with 1× PBS supplemented with 0.03% Triton X-100, and blocked with 1× PBS supplemented with 2% bovine serum albumin (BSA) and 5% normal horse serum. Primary antibodies were added and incubated at 4°C overnight in blocking buffer. Secondary Alexa-conjugated antibodies from Jackson ImmunoResearch Laboratories were added and incubated at room temperature for 1 h. The nuclei were counterstained with DAPI. The samples were imaged by laser confocal microscope (Olympus FV3000).

### 2.4. ELISA Analysis

The IL-1*β* levels in mouse peritoneal lavage fluids samples and cell culture supernatants were determined by ELISA kits (R&D Systems) following the manufacturer's instructions.

### 2.5. Immunoblotting Assay

The primary antibodies used in this study were listed by Supplementary materials. Mitochondria were isolated using mitochondria isolation kit (Beyotime, Shanghai, China, c3601). Whole cell lysates were prepared in RIPA buffer (25 mM Tris-HCl pH 7.6, 150 mM NaCl,1% NP-40, 1% sodium deoxycholate, 0.1% SDS) supplemented with a protease inhibitor cocktail (Roche). The protein concentrations were determined using a BCA Protein Assay Kit (Pierce, 23225). Equal amounts of protein were separated by SDS–PAGE and transferred to PVDF membranes. Then, the membranes were blocked with 5% BSA in 1× TBST for 1 h at room temperature and incubated with relevant antibodies overnight. The secondary antibodies were added and incubated for 1 h at room temperature, and the protein bands were detected using chemiluminescence reagents (Bio-Rad).

The ASC oligomer crosslinking assay was performed as previously described [24]. Cells were lysed with PBS supplemented with 0.5% Triton X-100 and a protease inhibitor cocktail (Roche). Cell lysates were centrifuged at 10,000 rpm for 15 min at 4°C. The supernatants were transferred to new tubes (TritonX-100 soluble fractions). The Triton X-100-insoluble pellets were washed with PBS three times and then suspended in 200 *μ*l of PBS. The pellets were then crosslinked by adding 2 mM disuccinimidyl suberate (DSS) (Thermo Fisher Scientific) at room temperature for 30 min. The crosslinked pellets were further centrifuged at 10,000 rpm for 15 min and directly dissolved in SDS sample buffer for SDS-PAGE.

### 2.6. Mitochondrial Function Detection

Mitochondrial morphology was observed by laser confocal microscope after MitoTracker Green probe staining. (Beyotime, Shanghai, China, C1048). Mitochondrial membrane potential was measured using a JC-1 probe (Beyotime, Shanghai, China, C2006). Mitochondrial reactive oxygen species (mtROS) production was measured using MitoSOX (Invitrogen) probe. Total intracellular ROS levels were measured using DHE (BestBio, BB-47051) probe. Cells were loaded with the corresponding probe and then washed three times. The cells were resuspended in PBS, and then the fluorescence intensity was measured with flow cytometry (FCM, NovoCyte 2060R) according to manufacturer's instructions. For laser confocal microscopy (LCM) assessment, DAPI was used for nuclear counterstaining, and the cells were washed three times with PBS. Images were obtained using laser confocal microscope (Olympus FV3000).

The oxygen consumption rate was measured using Seahorse XFe24 Extracellular Flux Assay Kits (Seahorse Bioscience, Billerica, MA, USA) as previously described [25]. BMDMs were cultured in Seahorse XFe24 cell culture microplates at a density of 2 × 10^5^ cells/well in DMEM supplemented with 10% FBS and antibiotics. After stimulation with MSU crystals (100 *μ*g/ml) for 12 h, the culture medium was replaced with unbuffered DMEM supplemented with 2 mMl glutamine, 10 mM glucose, and 2 mM pyruvate. The “Flux Pak” cartridge was hydrated with XF Calibrant Solution by overnight incubation in a non-CO_2_ incubator at 37°C. Oxygen consumption rate was measured, and the respiration rate was analyzed with step by step injections of mitochondrial complex inhibitors such as 1.5 *μ*M oligomycin A (56 *μ*l), 2 *μ*M FCCP (62 *μ*l), and 0.5 *μ*M rotenone–antimycinA cocktail (69 *μ*l) following the manufacturer's protocol. These mitochondrial complex inhibitors were provided with the Agilent Seahorse XF Cell Mito Stress Test Kit (Seahorse Bioscience, 103015-100). The data were normalized to total protein concentrations as measured using the Bradford assay. Data analysis was performed with the Seahorse Wave 2.2.0 software package (Seahorse Bioscience).

### 2.7. The mt-Keima Mitophagy Detection Assay

To develop stable expression cell lines, BMDMs were incubated with mt-Keima-COX8 lentiviruses (GENECHEM, Shanghai, China, LV01230-2a) at a multiplicity of infection (MOI) of 80, and polybrene was added to facilitate infection. At 24 h postinfection, surviving cells were selected with culture medium supplemented with puromycin (6 *μ*g/ml) to generate stable cell lines. BMDMs stably expressing mt-Keima-COX8 were treated with Mito-TEMPO or transfected with Drp1 siRNA, primed with LPS, and then stimulated with MSU crystals. For LCM detection, after the BMDMs were washed with PBS for three times, culture medium was added and LCM was used for imaging and analysis. The mitophagic ratio was calculated by measuring the red mean fluorescence intensity (MFI)/green mean fluorescence intensity ratio (excitation 561 : 488). For FCM detection, the cells were resuspended, and the cell suspensions were incubated on ice and measured with a flow cytometry (NovoCyte 2060R). Events were preselected for viable, single-cell populations, which were detected by excitation at 405 and emission at 610/620 nm. Fluorescent cells (20,000 per sample) were collected and analyzed for dual excitation at 488 (pH 7) and 561 (pH 4) nm with 582/515 and 610/620 nm emission filters, respectively. By analyzing the MFI (561) nm/MFI (488) nm ratio, the mitophagic ratio could be analyzed. Data processing was performed with FlowJo (v10, Tree Star) software.

### 2.8. MSU Crystal-Induced Peritonitis and Arthritis Mouse Models

C57BL/6 mice were intraperitoneally (ip) injected with Mito-TEMPO (20 mg/kg, Sigma–Aldrich, SML0737) or Mdivi-1(25 mg/kg, GLPBIO, GC10200), and after 1 h, the mice were injected with MSU suspension (3 mg in 100 *μ*L PBS). After 6 h, the mice were sacrificed under CO_2_ anesthesia for extraction of peritoneal fluid. Peritoneal lavage fluid was collected to measure the IL-1*β* levels by ELISA and to assess lymphocyte recruitment by FCM using the leukocyte common antigen FITC-CD45 (BD BioScience, 553079) staining, Pacific blue-CD11b (Biolegend, USA, 101245) costaining with PE-F4/80 (BD BioScience, 565410), and APC-Ly-6G (Biolegend 127614), respectively.

The C57BL/6 mice were intraperitoneally injected with Mito-TEMPO (20 mg/kg, Sigma-Aldrich, SML0737) or Mdivi-1(25 mg/kg, GLPBIO, GC10200) and then injected the foot pad with MSU suspension (1 mg in 50 *μ*L PBS). Twenty-four hours after injection with the MSU suspension, the swelling of the foot pad was measured with digital vernier caliper, and then the mice were sacrificed. Joint index evaluation was described previously [26].

### 2.9. Histological Evaluation and Immunohistochemistry

The pad tissue was removed from the mice and immediately fixed for 24 h, embedded in paraffin and sectioned. Hematoxylin and eosin (H&E) and immunofluorescence staining were performed. Details about the antibodies are provided in the supplementary materials. HE staining was used to evaluate the infiltration of inflammatory cells. For the immunofluorescence assay, briefly, foot pad sections were hydrated and boiled at 95°C in citrate buffer for 25 min. The sections were blocked with 2% BSA/PBS supplemented with 5% normal horse serum for 1 h at room temperature. Primary antibodies against MPO, CD11b, and DRP1 were incubated in blocking solution overnight at 4°C. After washing, Alexa secondary antibodies were added and incubated for 1 h at room temperature. The sections were washed with PBS and incubated with DAPI. The sections were imaged using laser confocal microscope.

### 2.10. Statistical Analysis

The values are expressed as mean ± SEM. Statistical analysis was performed using one-way ANOVA (analysis of variance). All the statistical analyses were assessed using the GraphPad Prism software (Version 6.01). Values were considered statistically significant when *P* < 0.05.

## 3. Results

### 3.1. Mito-TEMPO Improves Mitochondrial Dynamic Damage in MSU Crystal-Treated BMDMs

**Figure 1:**
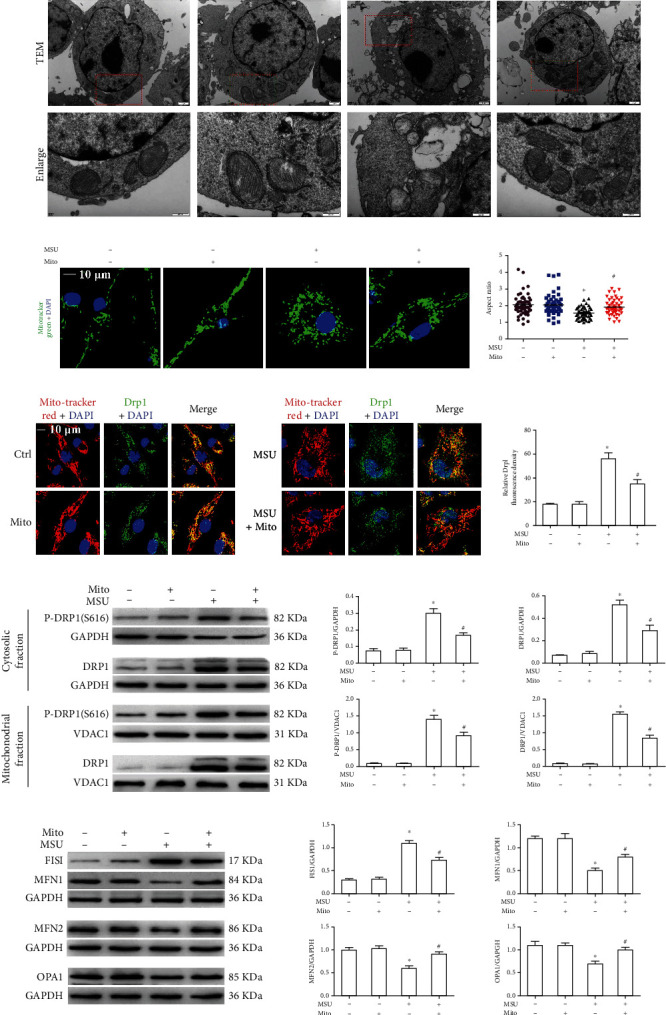
The effect of Mito-TEMPO on mitochondrial content and mitochondrial morphological changes induced by MSU crystals in BMDMs. BMDMs were pretreated with Mito-TEMPO (20 *μ*M, 14 h), primed with LPS (100 ng/ml, 1 h), and challenged with MSU suspension (75 *μ*g/ml, 12 h). (a) Mitochondria in the BMDMs were examined by TEM (*n* = 5). Scale bar: 0.5 *μ*m. (b) Representative LCM images of MitoTracker Green probe in live BMDM mitochondrial imaging and box and whisker plot of quantified mitochondrial aspect ratio; DAPI stains nuclei. Scale bars are 10 *μ*m, *n* = 3 experimental replicates with 100 mitochondria analyzed. (c) Representative LCM images of BMDMs costained with MitoTracker Red probe and DRP1 Ab. DAPI stains nuclei. Scale bars, 10 *μ*m. Experimental replicates with 100 mitochondria measured. (d) Immunoblot analysis was used to detect DRP1 and p-DRP1 in mitochondrial/cytosolic fractions. (e) Immunoblot analysis was used to detect Fis1, MFN1, MFN2, and OPA1 protein levels in cell lysates. All IB show one representative out of 3. ^∗^*P* < 0.05 vs. without MSU crystal treatment; ^#^*P* < 0.05 vs. MSU crystal treatment + vehicle.

Mitochondrial ROS can affect mitochondrial homeostasis. As mitochondria are the highly dynamic organelles, we analyzed whether Mito-TEMPO could affect mitochondrial dynamic balance in MSU crystal-treated BMDMs. By TEM, we detected the mitochondrial morphology in BMDMs. As shown in Figure 1(a), mitochondria were smaller and fragmented in BMDMs treated with MSU crystals. Mito-TEMPO treatment significantly alleviated mitochondrial fragmentation induced by MSU crystals. In addition, the mitochondrial morphology stained with MitoTracker Green (a probe that emits fluorescence) was assessed by laser confocal microscopy (LCM). A large number of small and round mitochondria was observed in BMDMs stimulated by MSU crystals. We also analyzed the aspect ratio (AR) of mitochondria. The mitochondrial AR analysis showed a decreasing trend in BMDMs stimulated with MSU crystals (Figure 1(b)), suggesting that MSU crystals accelerated mitochondrial fission. Mito-TEMPO successfully prevented mitochondrial morphological abnormalities in MSU crystal-treated BMDMs (Figure 1(b)). These data showed that Mito-TEMPO effectively relieved mitochondrial morphological damage induced by MSU crystals.

Disruption of mitochondrial dynamics affects mitochondrial morphology. Dynamin-related protein 1 (Drp1) is a known regulator of mitochondrial fission. The phosphorylation of Drp1 (p-Drp1) at Ser616 promotes Drp1 recruitment to mitochondria and subsequent mitochondrial fission [27]. Therefore, we investigated the effect of Mito-TEMPO on the mitochondrial distribution of MSU crystal-induced Drp1 protein. LCM imaging analysis revealed that MSU crystal exposure obviously promoted the elevation of Drp1 protein located in mitochondria, indicating the initiation of the fission pathway, and Mito-TEMPO pretreatment significantly reduced the fluorescence signal of Drp1 in mitochondria (Figure 1(c)). More importantly, western blot data showed that the expression of p-DRP1 (s616) in the mitochondrial fraction of BMDMs was decreased after Mito-TEMPO pretreatment (Figure 1(d)). We also explored the role of Mito-TEMPO on the mitochondrial fusion proteins (MFN1, MFN2, and OPA1) and mitochondrial fission protein Fis1 levels. Mito-TEMPO reversed the MSU crystal-induced increase in the Fis1 protein expression and the decrease in MFN1, MFN2, and OPA1 protein levels (Figure 1(e)). These data indicated that Mito-TEMPO might relieve mitochondrial dynamic damage by improving the imbalance of mitochondrial fission-fusion proteins.

### 3.2. Drp1 Signaling Is Involved in Mitochondrial Dysfunction

**Figure 2:**
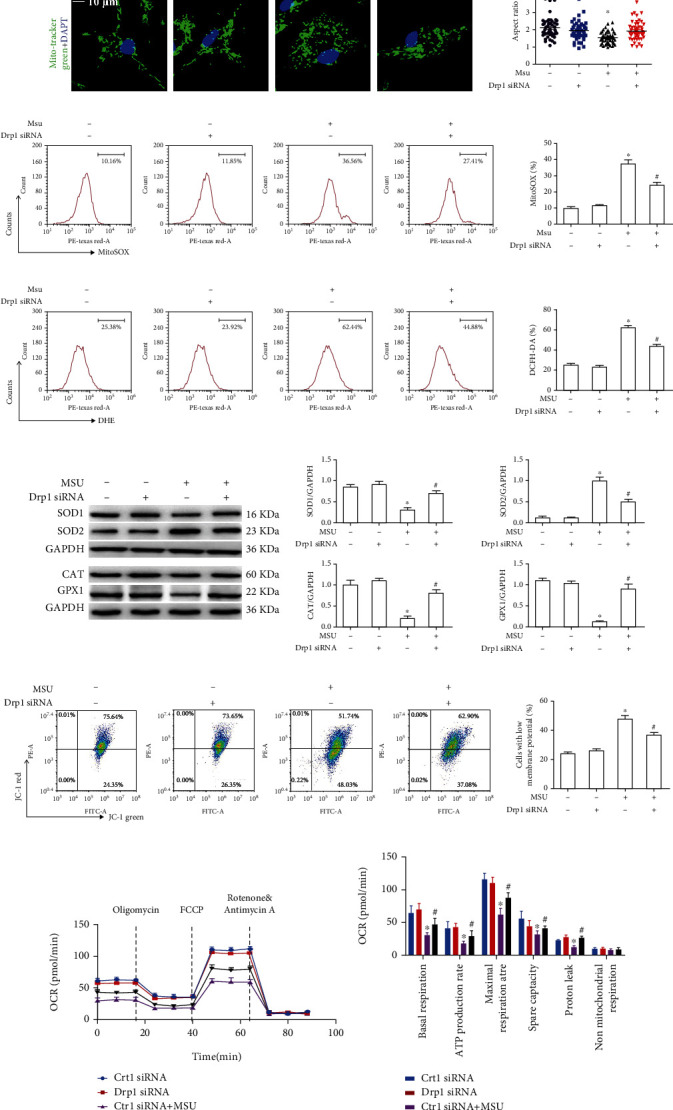
Drp1 knockdown mitigates MSU crystal-mediated mitochondrial dysfunction. (a) BMDMs were transfected with Drp1 siRNA or Ctrl siRNA for 48 h, and western blot was used to detect DRP1 protein level. (b)–(e) BMDMs were transfected with Drp1 siRNA or Ctrl siRNA for 48 h, primed with LPS (100 ng/ml, 1 h), and challenged with MSU suspension (75 *μ*g/ml). (b) Representative LCM images of MitoTracker Green probe in live BMDM mitochondrial imaging and box and whisker plot of quantified mitochondrial aspect ratio; DAPI stains nuclei. Scale bars are 10 *μ*m, *n* = 3 experimental replicates with 100 mitochondria analyzed. (c) Mitochondrial ROS levels were measured by FACS using MitoSOX probe. (d) Total intracellular ROS production was measured by FACS using DHE probes. (e) The protein levels of SOD1, SOD2, CAT, and GPX1 were detected by western blot. (f) JC-1 probe was used to detect MMP. (g) Oxygen consumption rate was measured using a Seahorse XFe24 Analyzer. (h) Bar graph showing individual mitochondrial function parameters calculated from data in the panel. ^∗^*P* < 0.05 vs. without MSU crystal treatment; ^#^*P* < 0.05 vs. MSU crystal treatment + NC siRNA.

We first detected the impact of Drp1 knockdown on mitochondrial morphology induced by MSU crystals. Drp1 was inhibited in BMDMs transfected with targeted Drp1 siRNA. (Figure 2(a)). The LCM imaging revealed that the enhanced mitochondrial aspect ratio in MSU crystal-treated BMDMs was also largely reversed by Drp1 knockdown (Figure 2(b)). Since the excessive mitochondrial fission mediated by Drp1 could exacerbate the production of mitochondrial ROS [28], we also observed that Drp1 knockdown significantly decreased mitochondrial ROS and total intracellular ROS generation in MSU crystal-treated BMDMs (Figures 2(c) and 2(d)). We sought to explore whether Drp1 knockdown mediated antioxidant protein expression induced by MSU crystals. SOD1, SOD2, CAT, and GPX1 are all intracellular antioxidant proteins. It is interesting to note that after MSU crystal stimulation, SOD1, CAT, and GPX1 protein levels decreased, while the protein levels of SOD2 increased. SOD2 is an important mitochondrial antioxidant enzyme residing in the mitochondrial matrix. In MSU crystal-induced BMDMs, Drp1 knockdown almost restored the expression of these antioxidant proteins (Figure 2(e)). These data suggested that there was crosstalk between mitochondrial ROS and Drp1 signaling in MSU crystal-induced inflammation. Excessive ROS levels usually cause the *Δψ*m to collapse by disrupting mitochondrial membrane potential homeostasis. The loss of mitochondrial membrane potential induced by MSU crystals was partially recovered in Drp1 knockdown BMDMs (Figure 2(f)).

Drp1 is involved in regulating mitochondrial dynamics induced by MSU crystals, and mitochondrial dynamics play an important role in the response of cells to exogenous stress. In this study, mitochondrial stress was assessed by monitoring the real-time oxygen consumption rate (OCR) using the Seahorse XFe24 Extracellular Flux Analyzer. Interestingly, mitochondrial basal respiration, ATP production, maximal respiration rate, spare capacity, and proton leak were significantly decreased in the BMDMs exposed to MSU crystals (Figures 2(g) and 2(h)). Drp1 knockdown significantly attenuated the mitochondrial stress induced by MSU crystals (Figures 2(g) and 2(h)). Furthermore, the Drp1 overexpression exacerbated the mitochondrial stress induced by MSU crystals (Supplementary Figures 2(a)-2(b)).

### 3.3. MSU Crystals Promote Mitophagy Initiation and Increase Mitolysosome Formation

**Figure 3:**
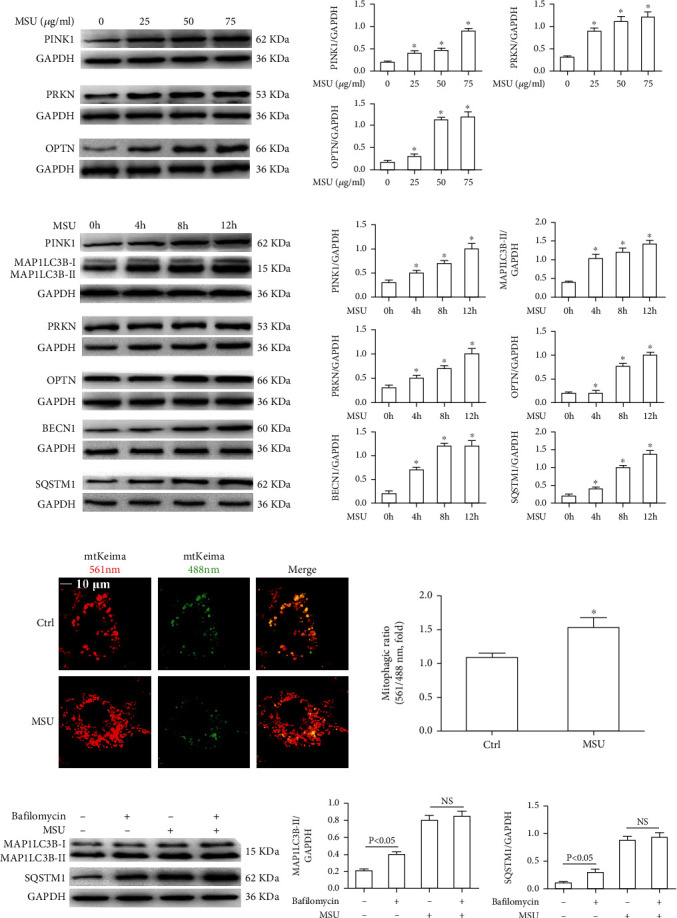
MSU crystals accelerate the initiation of mitophagy and upregulate the formation of mitolysosomes. (a) The protein levels of PINK1, PRKN, and OPTN were detected by immunoblot in BMDMs primed with LPS (100 ng/ml, 1 h) and treated with MSU crystals (25, 50, 75 *μ*g/ml) at different concentrations for 12 h. (b) Immunoblotting was performed on PINK1, PRKN, OPTN, BECN1, MAP1LC3B-II, and SQSTM1 protein levels in BMDMs primed with LPS (100 ng/ml, 1 h) and treated with MSU crystals (75 *μ*g/ml) for different time points (4 h, 8 h, 12 h). (c) The mt-Keima assay was employed to assess mitophagy in BMDMs primed with LPS (100 ng/ml, 1 h) and treated with MSU crystals (75 *μ*g/ml) for 12 h. The fluorescence intensity ratio of 561/488 nm was used to quantify mitophagy index. Scale bars are 10 *μ*m, *n* = 3 experimental replicates with 100 cells analyzed. (d) Western blot analysis and the expression of MAP1LC3B-II and SQSTM1 in BMDMs primed with LPS (100 ng/ml, 1 h) and exposed to MSU crystals (75 *μ*g/ml) for 12 h followed by treatment with 400 nM bafilomycin A1, added during the last 4 h of the 12 h treatment period. Data are shown as *mean* ± *SEM*. All IB show one representative out of 3. ^∗^*P* < 0.05 vs. without MSU crystal treatment, ^#^*P* < 0.05 vs. MSU crystal treatment.

MSU crystal stimulation results in mitochondrial damage, which requires clearance through mitophagy. Next, we attempted to detect the effect of MSU crystal exposure on the initiation of mitophagy. The protein expression of mitophagy markers, such as PINK1, PRKN, and OPTN, was evaluated using immunoblotting of protein lysates from BMDMs stimulated with different doses of MSU crystals (25, 50 and 75 *μ*g/ml) for 12 h. Interestingly, MSU crystals almost dose-dependently promoted the expression of PINK1, PRKN, and OPTN (Figure 3(a)). Next, we performed a time-course experiment to determine the optimal time point at which MSU crystal-induced upregulation of the mitophagy marker protein expression in BMDMs is as follows. BMDMs were stimulated with 75 *μ*g/ml MSU crystals for the indicated time points (0, 4, 8, 12 h), and cell lysates were used to measure the mitophagy marker protein expression by immunoblotting. The PINK1 protein expression almost reached its peak 8 h after exposure to MSU crystals, while PRKN and OPTN protein levels peaked at approximately 12 h (Figure 3(b)).

In the process of mitophagy, depolarized mitochondria cannot import and degrade PINK1, which is activated by the PRKN protein and modified by the OPTN protein, finally resulting in its elimination via the BECN1/MAP1LC3B-dependent autophagy process. Next, we aimed to monitor the expression of proteins involved in the autophagy process, including autophagosome initiation marker (BECN1), autophagosome formation marker (MAP1LC3B), and autophagy degradation marker (SQSTM1) in BMDMs stimulated with MSU crystals (75 *μ*g/ml) for varying time points. The expression of the autophagy markers BECN1, MAP1LC3B-II, and SQSTM1 was significantly increased in BMDMs exposed to MSU crystals over a period of up to 12 hours (Figure 3(b)).

Next, we measured functional mitophagy using a mitochondrial targeted mt-Keima probe, which is an indicator of mitochondria colocalization with mature autolysosomes. Since the mt-Keima probe is resistant to lysosomal proteases and exhibits reversible color changes at acidic pH levels, the mt-Keima probe can be used to assess autolysosome maturation. The high excitation peak ratio (561: 488) of mt-Keima is shown in pseudo, indicating the presence of mitochondria in mature autolysosomes. Using this assay, we observed that the number of red mt-Keima puncta was significantly increased in BMDMs exposed to MSU crystals, indicating an increased level of mitolysosomes (Figure 3(c)). Bafilomycin A1 is a known inhibitor of the late phase of autophagy and can inhibit the fusion of phagosomes and lysosomes. As shown in Figure 3(d), in non-MSU crystal treatment BMDMs, the levels of LC3B-II and SQSTM1 were significantly higher after the application of bafilomycin A1 (Figure 3(d)). Nevertheless, the levels of LC3B-II and SQSTM1 were not further increased by the application of bafilomycin A1 in the BMDMs treated with MSU crystals, compared with MSU crystal-induced cells (Figure 3(d)). All these findings suggest that MSU crystals might accelerate the fusion of mitophagosmes and lysosomes to form mitolysosomes, but autophagic flux is blocked in MSU crystal-induced BMDMs.

### 3.4. Silence of Drp1 Expression Rescued Defective Mitophagy Induced by MSU Crystals

**Figure 4:**
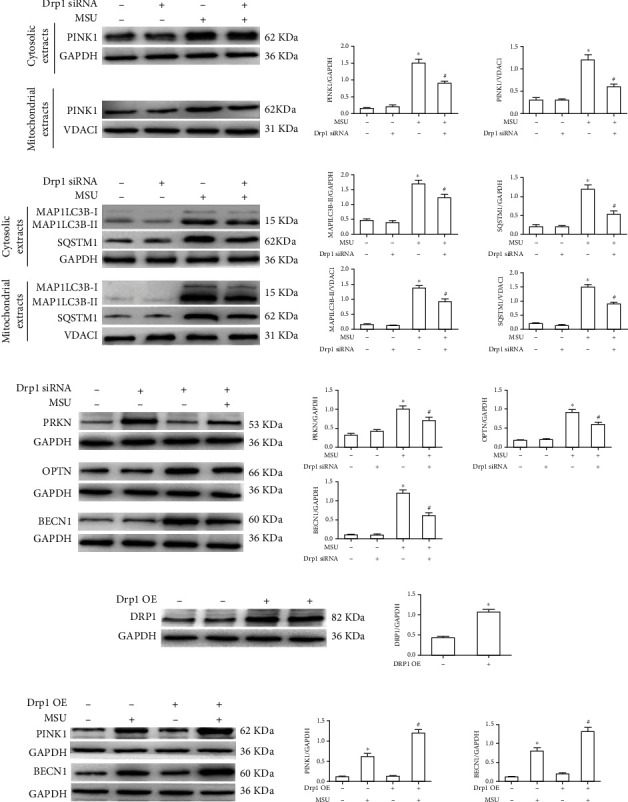
Drp1 signaling affected mito/autophagy marker protein levels induced by MSU crystals. (a, b) BMDMs were transfected with either Drp1 siRNA or Ctrl siRNA for 48 h, then primed with LPS (100 ng/ml, 1 h), and stimulated with MSU suspension (75 *μ*g/ml, 12 h). (a) For PINK1, MAP1LC3B, and SQSTM1, BMDMs were performed mitochondrial/cytosolic fractionation. Immunoblot analysis was used to detect PINK1, MAP1LC3B, and SQSTM1 in mitochondrial/cytosolic fractions. (b) PRKN, OPTN, and BECN1 protein levels were also detected in cell lysates. (c) BMDMs were transfected with either Ctrl plasmid or Drp1 plasmid for 48 h, and western blot was used to detect DRP1 protein levels. (d) Immune blotting was used to measured DRP1, PINK1, and BECN1 protein levels in Drp-1 overexpressed BMDMs that were primed with LPS (100 ng/ml, 1 h) and challenged with MSU suspension.

In the present study, we further investigate the role of Drp1 in MSU crystal-induced mitophagy response. As expected, the immunoblotting data of mitochondrial extracts showed that the PINK1, MAP1LC3B-II, and SQSTM1 proteins were highly expressed in MSU crystal-treated BMDMs, while Drp1 knockdown significantly reduced their accumulation in mitochondria (Figure 4(a)). Drp1 knockdown also greatly inhibited MSU crystal-induced expression of PRKN, OPTN, and BECN1 (Figure 4(b)). Drp1 was overexpressed in BMDMs transfected with corresponding plasmid. (Figure 4(c)). However, the Drp1 overexpression in BMDMs aggravated the expression of mito/autophagy markers PINK1 and BECN1 induced by MSU crystals (Figure 4(d)).

**Figure 5:**
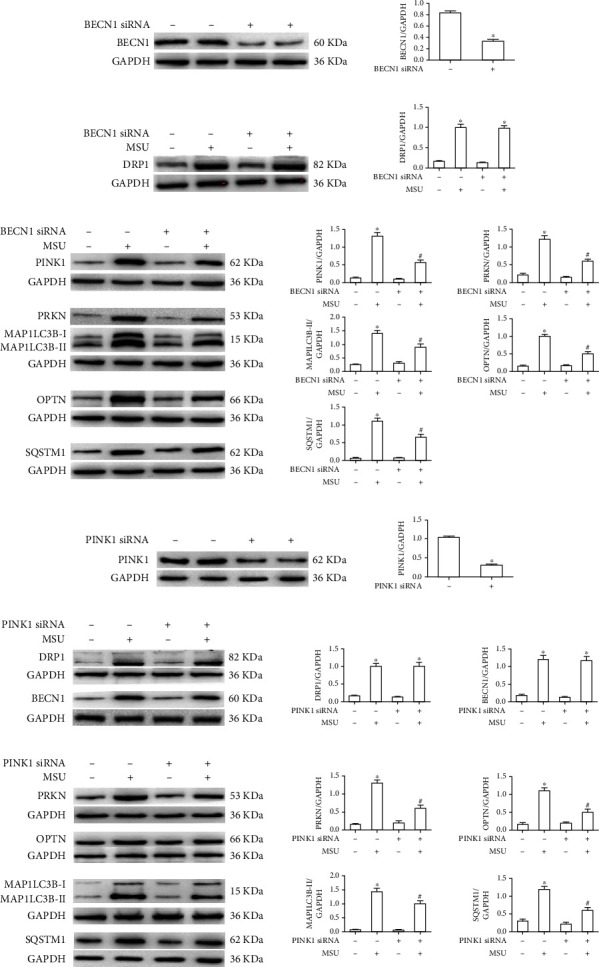
The effect of BECN1 or PINK1 knockdown on Drp1 and auto/mitophagy marker protein levels. (a) BMDMs were transfected with BECN1 siRNA or Ctrl siRNA for 48 h, and western blot was used to detect BECN1 protein level. (b, c) BECN1, DRP1, PINK1, PRKN, MAPILC3B-II, OPTN, and SQSTM1 protein levels were analyzed by immunoblotting in BECN1 knockdown BMDMs that were primed with LPS and challenged with MSU suspension. (d) BMDMs were transfected with PINK1 siRNA or Ctrl siRNA for 48 h, and western blot was used to detect PINK1 protein level. (e, f) PINK1, DRP1, BECN1, PRKN, OPTN, MAPILC3B-II, and SQSTM1 protein levels were analyzed by immunoblotting in PINK1 knockdown BMDMs that were primed with LPS and challenged with MSU suspension. All WB show one representative out of 3. ^∗^*P* < 0.05 vs. without MSU crystal treatment; ^#^*P* < 0.05 vs. MSU crystal treatment + vehicle.

Next, we attempted to genetically block autophagy to confirm the role of this process in MSU crystal-induced mitophagy. BMDMs were transfected with either BECN1 siRNA or Ctrl siRNA (Figure 5(a)). DRP1 and mito/autophagy marker protein levels were measured by immunoblotting. Downregulation of BECN1 almost had no effect of DRP1 protein level induced by MSU crystals (Figure 5(b)). However, the MSU crystal-induced upregulation of mito/autophagy marker protein (including PINK1, PRKN, OPTN, MAP1LC3B-II and SQSTM1) expression was prevented in BMDMs transfected with BECN1 siRNA (Figure 5(c)). The effect of mitophagy initiation signaling on the expression of DRP1 and mito/autophagy marker proteins was also detected. BMDMs were transfected with either Ctrl siRNA or PINK1 siRNA (Figure 5(d)). Further western blot data showed that PINK1 knockdown had little effect on the MSU crystal-mediated DRP1 protein levels and the autophagy initiation marker BECN1 (Figure 5(e)) but prevented the upregulation of the mitophagy markers (PRKN and OPTN) and the autophagy markers (MAP1LC3B-II and SQSTM1) (Figure 5(f)). These data imply that Drp1 signaling is upstream of autophagy initiation and PINK1/PRKN-mediated priming of damaged mitochondria.

**Figure 6:**
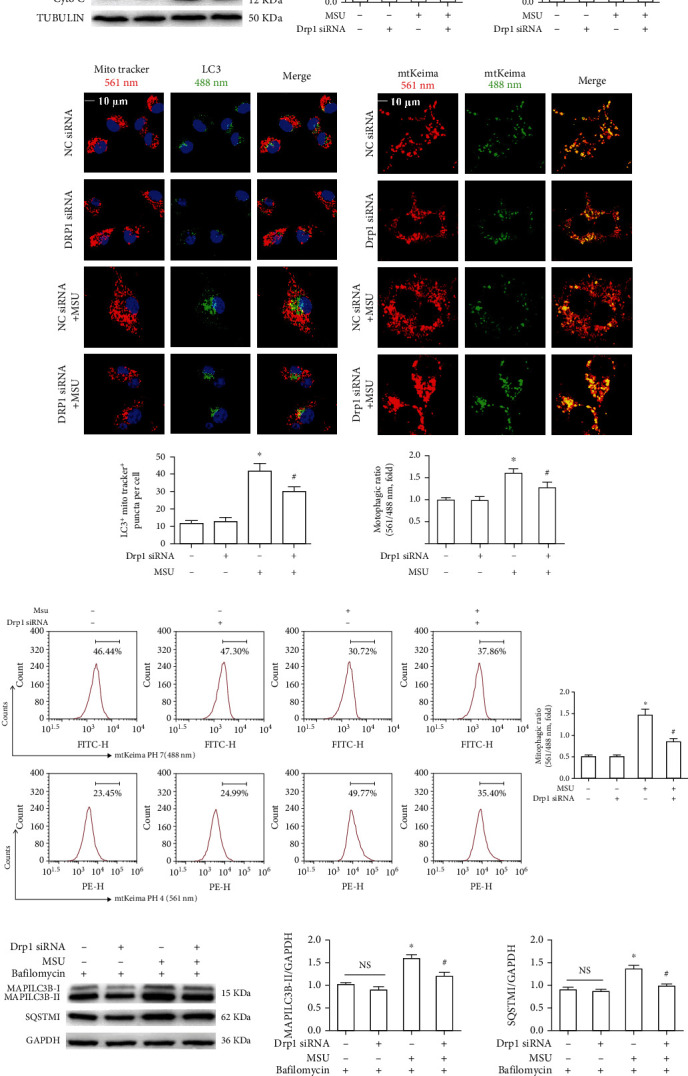
Drp1 knockdown alleviated the accumulation of mitochondrial marker protein, mitophagosomes, and mitolysosomes induced by MSU crystals. (a)–(d) BMDMs were transfected with either Drp1 siRNA or Ctrl siRNA for 48 h, then primed with LPS (100 ng/ml, 1 h), and stimulated with MSU suspension (75 *μ*g/ml, 12 h). (a) Western blot was used to detect TOM20 and Cyto C protein levels. (b) BMDMs were subjected to immunofluorescence staining of LC3 (green) and MitoTracker Deep Red (red). (c, d) After BMDMs stably expressing mt-Keima-COX8 plasmid, transfected with Drp1 siRNA or negative control (NC) siRNA for 48 h, primed with LPS (100 ng/ml, 1 h), and then stimulated with MSU crystals (75 *μ*g/ml) for 12 h. (c) Representative LCM images of mitolysosome formation. The mean fluorescence intensity ratio of 561/488 nm was used to quantify mitophagic ratio. Scale bars are 10 *μ*m, *n* = 3 experimental replicates with 100 cells analyzed. (d) FCM experiments and analysis were used to analysis mitophagic ratio. (e) Representative western blots showing the expression of MAP1LC3B-II and SQSTM1 in BMDMs transfected with Drp1 siRNA or negative control (NC) siRNA for 48 h, primed with LPS (100 ng/ml, 1 h), and exposed to MSU crystals (75 *μ*g/ml) for 12 h followed by treatment with 400 nM bafilomycin A1, added during the last 4 h of the 14 h treatment period. Experiments were repeated for at least three times, and data are shown as mean ± SEM from 3 independent experiments. All IB show one representative out of 3. ^∗^*P* < 0.05 vs. without MSU crystal treatment; ^#^*P* < 0.05 vs. MSU crystal treatment + vehicle.

Subsequently, we further explored the effect of Drp1 knockdown on the mitophagic response through multiple assays. First, we detected the impact of Drp1 knockdown on the expression of the mitochondrial marker proteins Tom20 and Cyto C in BMDMs treated with MSU crystals. They are commonly used as mitochondrial markers to reflect damage mitochondrial clearance and mitophagic rate [29]. Silence of Drp1 alleviated the elevation of Tom20 and Cyto C protein levels induced by MSU crystals (Figure 6(a)). Second, we examined mitophagy by costaining mitochondria (labeled by MitoTracker Red) and LC3 (an autophagy marker) to reflect formation of mitophagosomes. The immunofluorescence staining showed that Drp1 knockdown decreased colocalization of LC3^+^ puncta with MitoTracker in BMDMs treated with MSU crystals (Figure 6(b)), indicating that silence of Drp1 inhibited the accumulation of damaged mitochondria and mitophagosomes. Third, we used the mitophagy reporter Mito-Keima to quantitatively detect mitophagic activity. After the stable expression of Mito-Keima-COX8 plasmid by BMDMs, interfered with Drp1 siRNA or NC siRNA, and then stimulated with MSU crystals, LCM imaging data and flow cytometry analysis, respectively, indicated that NC siRNA and Drp1 siRNA BMDMs almost exhibited similar mitophagy at baseline level without stimulation of MSU crystals, as reflected by similar percentage of the cells with a high 561/405 nM ratio (Figures 6(c) and 6(d)). As expected, downregulation of Drp1 markedly decreased the percentage of cells with the high 561/488 nm ratio in BMDMs treated with MSU crystals (Figures 6(c) and 6(d)). Fourth, we monitored autophagic rate to analyze LC3-II and P62 protein levels by adding autophagosome (mitophagosome) and lysosome fusion inhibitor (bafilomycin A1). Western blot data indicated that Drp1 knockdown in the presence of MSU crystals effectively prevented the accumulation of LC3-II and P62 in BMDMs treated with bafilomycin A1 (Figure 6(e)). Taken together, these findings suggest that silence of Drp1 effectively relieved impaired mitophagy induced by MSU crystals.

### 3.5. Drp1 Knockdown Relieved NLRP3 Inflammasome Activation Induced by MSU Crystals

**Figure 7:**
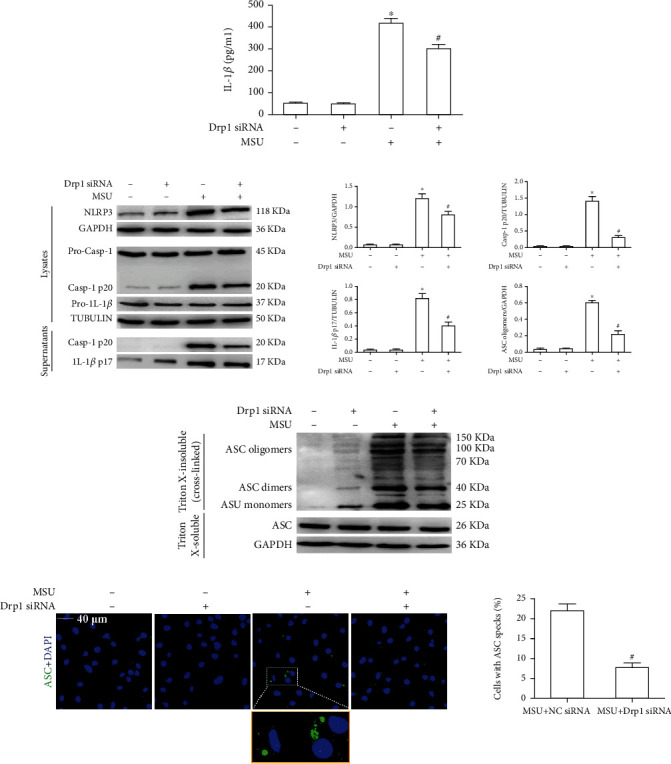
Drp1 knockdown relieved MSU crystal-induced NLRP3 inflammasome activation. (a)–(d) BMDMs were transfected with Drp1 siRNA or Ctrl siRNA, primed with LPS (100 ng/ml, 1 h), and challenged with MSU suspension (75 *μ*g/ml, 12 h). (a) Secretion of IL-1*β* was detected by ELISA in the culture supernatants. (b) NLRP3, caspase-1 (pro form and cleaved p20), and IL-1*β* (pro form) expression was assessed by western blotting. Mature IL-1*β* and the p20 fragment of caspase-1 were also detected in cell culture supernatants. Tubulin was used as the loading control. (c) Total cell lysates were obtained in Triton X-100–containing buffer. Insoluble (pellet) fractions were crosslinked with DSS to capture ASC oligomers. The soluble and insoluble fractions were analyzed by immunoblotting with an ASC antibody (Ab). GAPDH was used as a loading control. Blots are representative of 3 independent experiments. (d) Representative LCM images of BMDMs stain with ASC Ab. DAPI stains nuclei. Arrows indicate ASC specks. Scale bars, 10 *μ*m. Results are averages ± SEM (*n* = 3). ^∗^*P* < 0.05 vs. without MSU crystal treatment; ^#^*P* < 0.05 vs. MSU crystal treatment + vehicle.

Since Drp1 signaling is involved in mitochondrial fitness and mitophagy, next, we investigated whether Drp1 knockdown ameliorated NLRP3 inflammasome activation induced by MSU crystals. Drp1 knockdown inhibited IL-1*β* secretion (Figure 7(a)) and moderately decreased the expression of NLRP3 which is the important component of NLRP3 inflammasome, but Drp1 knockdown had almost no effect on the protein expression of pro-IL-1*β* and pro-caspase-1 in LPS-primed BMDMs treated with MSU crystals (Figure 7(b)). Importantly, Drp1 knockdown blunted Ccspase-1 activation and pro-IL-1*β* processing into mature IL-1*β* in LPS-primed BMDMs incubated with MSU crystals (Figure 7(b)). ASC oligomerization leading to ASC speck formation mediates caspase-1 activation [30, 31]. The effect of Drp1 knockdown on ASC oligomerization and subsequent ASC speck formation was also analyzed. ASC oligomerization was measured by immunoblotting analysis of the DSS–crosslinked insoluble BMDM lysate fraction. In LPS-primed BMDMs treated with MSU crystals, Drp1 knockdown blocked ASC oligomerization (Figure 7(c)), and similar results were obtained by immunofluorescence staining of ASC speck information (Figure 7(d)).

### 3.6. Silence of PINK1 or BECN1 Fails to Suppress Mitochondrial Damage in MSU Crystal-Treated BMDMs

**Figure 8:**
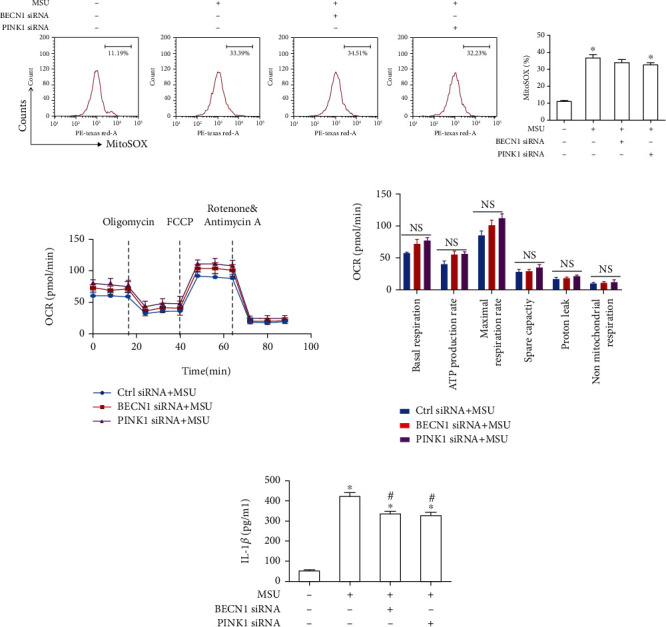
PINK1 or BECN1 knockdown fails to affect mitochondrial dysfunction in MSU crystal-treated BMDMs. (a)–(c) BMDMs were transfected with either PINK1 siRNA or Ctrl siRNA for 48 h, then primed with LPS (100 ng/ml, 1 h) and stimulated with MSU suspension (75 *μ*g/ml, 12 h). (a) Mitochondrial ROS levels were measured by FACS using MitoSOX probe. (b) Oxygen consumption rate was measured using a Seahorse XFe24 Analyzer. Bar graphs show the relative parameters of the mitochondrial respiratory function. (c) The level of IL-1*β* was detected by ELISA in the culture supernatants. The data are presented as mean ± SEM from 3 independent experiments. ^∗^*P* < 0.05 vs. without MSU crystal treatment + Ctrl siRNA; ^#^*P* < 0.05 vs. MSU crystal treatment + vehicle.

We attempted to study the role of auto/mitophagy in mitochondrial function. To this end, BMDMs were transfected with PINK1 or BECN1 siRNA to knock down its expression, and mitochondrial function was estimated. Our findings demonstrated that transfection of BMDMs with either PINK1 or BECN1 siRNA had little effect on the MSU crystal-mediated elevation of mitochondrial ROS production (Figure 8(a)) and decrease in the OCR (Figure 8(b)), suggesting that MSU crystal-mediated mitochondrial dysfunction was upstream of the mitophagy process. Blockage of mitophagy can exacerbate inflammation [19–21]. MSU crystal exposure induced IL-1*β* secretion, which was decreased after silence of BECN1 or PINK1 (Figure 8(c)), suggesting that BECN1 or PINK1 might be involved in NLRP3 inflammasome activation induced by MSU crystals.

### 3.7. Inhibition of Mitochondrial ROS or Drp1 Activity Alleviates MSU Crystal-Induced Inflammatory Response In Vivo

In light of the important role of mitochondrial ROS and Drp1 activity in the MSU crystal-induced inflammation in vitro, we studied whether ROS and Drp1 signaling are involved in MSU crystal-induced mouse arthritis and peritonitis models. Mouse models of arthritis and peritonitis were established by injecting MSU crystals into the foot pad and the abdominal cavity, respectively, and the mice were pretreated with Mito-TEMPO or Mdivi-1 by intraperitoneal injection.

**Figure 9:**
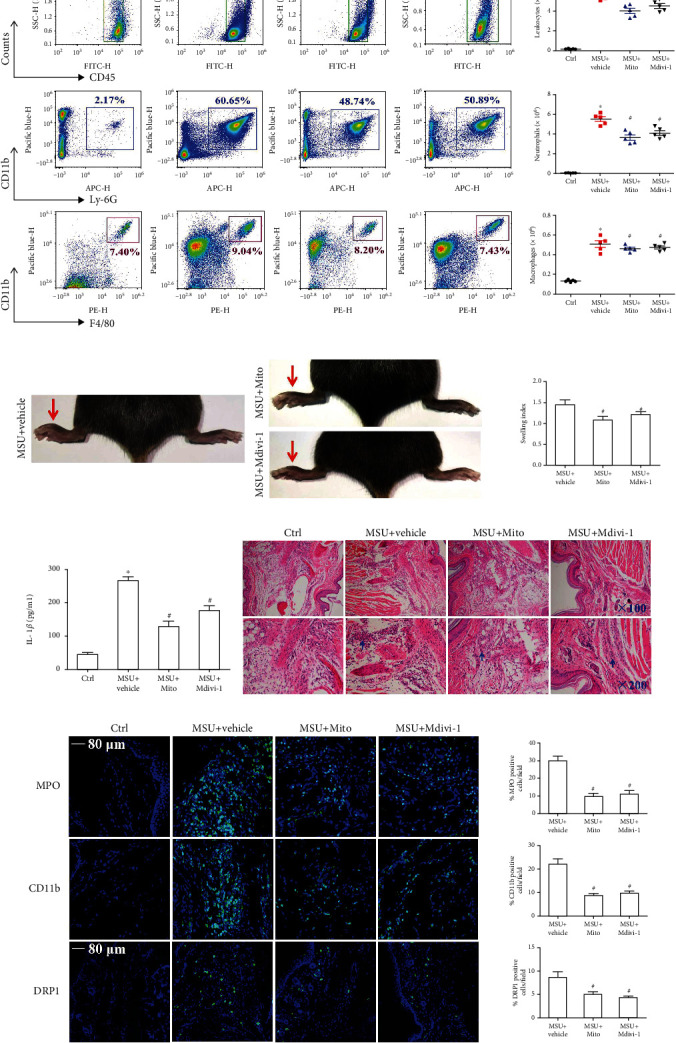
Mito-TEMPO or Mdivi-1 pretreatment on MSU crystal-induced mouse inflammatory model produced protective effects. (a) Representative plots of migrated leukocytes (CD45^+^), neutrophils (CD11b^+^Gr-1^+^), and macrophages (CD11b^+^F4/80^+^) in peritoneal lavage fluid were detected by FCM. The number of migrated leukocytes, neutrophils, and macrophages were quantified and compared among the groups. (b) Paw swelling index. (c) The supernatants of peritoneal fluid were checked by ELISA for IL-1*β*. (d) HE staining of mouse footpad tissue section and representative image of microscope. *n* = 5 for each group. (e) An immune-fluorescence assay was used to detect MPO, CD11b, and DRP1-positive cell distribution in mouse foot pad tissue sections. Blue shows nuclei stained with DAPI. *n* = 5 mice per group. 10–12 images per mouse were analyzed. Data is average ± SEM. ^∗^*P* < 0.05 vs. Ctrl; ^#^*P* < 0.05 vs. MSU crystals + vehicle. All the data are expressed as the means ± SEM.

A peritonitis model in C57BL/6 mice was used to assess the role of Mito-TEMPO or Mdivi-1 on inflammatory cell influx and IL-1*β* production. As shown in Figure 9(a), MSU crystals accelerated the infiltration of leukocytes (CD45^+^), macrophages (CD11b^+^ F4/80^+^), and neutrophils (CD11b^+^ Ly-6G^+^) into the peritoneal cavity. The number of leukocytes, macrophages, and neutrophils was greatly diminished after Mito-TEMPO or Mdivi-1 pretreatment (Figure 9(a)). In contrast with vehicle treatment, Mito-TEMPO or Mdivi-1 pretreatment also obviously suppressed the MSU crystal-induced IL-1*β* secretion in peritoneal lavage fluids (Figure 9(c)). However, we noted that Mdivi-1 treatment had no significant difference in inflammatory cell migration and IL-1*β* secretion in the peritonitis model compared with Mito-TEMPO treatment.

Injection of a certain dose of MSU suspensions into the mouse foot pad can also lead to inflammatory response. Our data showed significant reduction in MSU crystal-induced foot pad swelling after Mito-TEMPO or Mdivi-1 pretreatment (Figure 9(b)). H&E staining showed that there were a large number of immune cells infiltrated into the tissue sections of the foot pad injected with the MSU suspensions, but Mito-TEMPO or Mdivi-1 administration noticeably reduced immune cell infiltration (Figure 9(d)). After immunofluorescence staining of foot pad tissue sections, LCM imaging data showed that Mito-TEMPO or Mdivi-1 pretreatment reduced the distribution of inflammatory cell (MPO and CD11b^+^-positive cells) (Figure 9(e)). We also noted that almost no DRP1 positive cells were distributed in the foot pad of Ctrl mice, while a number of DRP1-positive cells infiltrated the foot pad in MSU suspension treated mice, Mito-TEMPO or Mdivi-1 pretreatment decreased the number of DRP1-positive cells in foot pad of MSU suspension treated mice (Figure 9(e)). This suggested that in vivo MSU treatment promoted Drp1 expression, and Mito-TEMPO or Mdivi-1 could reduce Drp1 protein levels. Thus, all these data indicated that pretreatment with Mito-TEMPO or Mdivi-1 notably relieved the inflammation induced by MSU crystals in vivo, and that targeting mitochondrial ROS or Drp1 activity may be potential candidates for the treatment of gouty arthritis in the future.

## 4. Discussion

Previous work has documented the role of oxidative stress and mitochondrial dysfunction in inflammatory responses induced by MSU crystals. However, the molecular mechanism of mitochondrial ROS involvement in MSU crystal-induced inflammatory response is not fully understood. Although crosstalk between Drp1 signaling and ROS has been reported to be involved in some pathological processes [32–34], it is unclear whether there is a bidirectional relationship between Drp1 signaling and ROS in MSU crystal-induced inflammation.

In the current study, after Mito-TEMPO treatment, MSU crystal-induced upregulation of DRP1 and p-DRP1 (ser616) protein expression in mitochondria was effectively inhibited. Drp1 knockdown significantly alleviated the excessive ROS production induced by MSU crystals. These findings suggest that there is a bidirectional relationship between mitochondrial ROS and Drp1 signaling in MSU crystal-induced inflammation. Furthermore, we found that MSU crystals disrupted mitochondrial fission-fusion balance and led to mitochondrial morphological damage. Drp1 plays a key role in mediating the dynamic balance of mitochondrial fission-fusion [35, 36]. The phosphorylation of DRP1 at ser616 affects its activity and promotes mitochondrial fission [37]. MSU crystal treatment upregulated the expression of DRP1 and p-DRP1 (ser616) and especially promote the recruitment of P-DRP1 (ser616) to mitochondria, which is a key step in activating DRP1 GTPase of mitochondrial fission. Drp1 knockdown significantly reversed the increase in mitochondrial fragmentation induced by MSU crystals. These data imply that Drp1 signaling affects mitochondrial fission and maintains mitochondrial dynamic homeostasis in MSU crystal-induced inflammation.

MSU crystals promote excessive ROS generation leading to mitochondrial damage. Mitophagy is an important mitochondrial quality control mechanism that eliminates damaged mitochondria. In the process of mitophagy, damaged mitochondria are primed, sequestered, and removed via lysosomal degradation [38, 39]. PINK1 and PRKN are key components of the mitochondrial quality control system [40, 41]. Mitochondrial priming is initiated by PINK1 and PRKN, which are subsequently recruited to damaged mitochondria. Within depolarized mitochondria, PINK1 accumulation can recruit PRKN from the cytoplasm to the outer mitochondrial membrane initiating mitophagy. OPTN, a cytoplasmic adaptor protein, is an important receptor for mitophagy [42]. Although studies have reported that MSU crystals can affect the expression of autophagy or mitophagy–related proteins [43–46], the time- and dose-dependent effects of MSU crystals on the expression of autophagy and mitophagy marker proteins have not been reported in BMDMs. Our study showed that the expression of mitophagy marker proteins (PINK1, PRKN, and OPTN) and autophagy-related proteins (BECN1, MAP1LC3B-II, and SQSTM1) was significantly elevated in a time- or dose-dependent manner after exposure of BMDMs to MSU crystals. These data indicate that MSU crystal stimulation initiates the mitophagy pathway in BMDMs. After the induction of mitophagy, the mitophagosomes that formed are continually degraded by lysosomes via the lysosomal fusion pathway. We further confirmed that MSU crystals accelerated the formation and accumulation of mitolysosomes in BMDMs through the mt-Keima assay. The aggregation of MAP1LC3B-II and SQSTM1 induced by MSU crystal was not significantly affected by bafilomycin A1 treatment. These findings reveal that although MSU crystals promote the formation of mitolysosomes, the mitophagic degradation might be defective.

Mitochondrial dynamics also play an important role in mitophagy, and the steady-state imbalance of fission-fusion dynamics is an important cause of mitophagy. In addition, mitochondrial fission produces asymmetric daughter mitochondria. The ones with normal membrane potential can serve for mitochondrial biogenesis, while unhealthy daughter mitochondria with loss of membrane potential can be degraded by mitophagy [47]. It has been reported that ROS can damage lysosomal membrane stability [48] and then affect autophagic function. We observed that Drp1 could regulate both ROS levels and mitochondrial dynamics in BMDMs treated with MSU crystals. Drp1 inhibition can attenuate PINK1/Parkin-mediated mitophagy and increase Parkin recruitment to mitochondria and the rate of mitophagy [49, 50]. In the study, downregulation of Drp1 decreased levels of mito/autophagy marker protein induced by MSU crystals. Annadurai et al. reported that PINK1 and BECN1 are located upstream of the DRP1 signaling in cocaine-mediated mitophagy [30]. Our study indicated that gene silencing of BECN1 or PINK almost did not abrogate the MSU crystal-mediated elevation of Drp1 protein expression and mitochondrial stress. In the pathological processes, Drp1 is involved in different signaling pathways. BECN1 knockdown significantly inhibited the MSU crystal-induced upregulation of PINK1/PRKN, but downregulation of PINK1 almost had no effect on the MSU crystal-mediated elevation of BECN1 protein expression, indicating that the initiation of autophagy could regulate the priming of damaged mitochondria. Drp1 knockdown decreased MSU crystal-induced mitochondrial marker protein levels and accumulation of mitophagosome and mitolysosome. We speculate that Drp1 signaling not only regulates the priming of damaged mitochondria but also plays a role in mitophagosome formation and mitolysosome degradation.

Mitophagy can mitigate the activation of the NLRP3 inflammasome by regulating mitochondrial quality, maintaining mitochondrial homeostasis and eliminating damaged mitochondria [51–53]. Defective mitophagy accelerates the activation of the NLRP3 inflammasome, which may lead to metabolic and autoinflammatory diseases [29, 54]. Mito/autophagy is also involved in MSU crystal-induced NLRP3 inflammasome activation or IL-1*β* release [54]. Drp1-mediated excessive mitochondrial fission can promote the activation of the NLRP3 inflammasome [28]. However, the role of Drp1 signaling on NLRP3 inflammasome activation induced by MSU crystals is ambiguous. Drp1 knockdown could also effectively block the NLRP3 inflammasome activation in MSU crystal-treated BMDMs. So, we assume that Drp1 signaling regulates both mitochondrial damage and clearance of damaged mitochondria, thereby affecting NLRP3 inflammasome activity. It is important to find low-toxicity and high-efficiency small molecules that target the Drp1 expression to alleviate the inflammatory response induced by MSU crystals. Previous study has reported that inhibition of NLRP3 inflammasome activation can improve the mitochondrial mechanisms and pathway [55, 56]. We still need to further investigate the effect of inhibiting NLRP3 inflammasome activation on mitochondrial function.

In conclusion, our study revealed crosstalk between mitochondrial ROS and Drp1 signaling in MSU crystal-induced inflammation. Drp1 knockdown improved MSU crystal-induced mitochondrial damage, defective mitophagy, and the NLRP3 inflammasome activation. In vitro and in vivo Drp1 inhibition significantly ameliorated the MSU crystal-induced inflammatory response.

## Data Availability

The datasets used and/or analyzed during the present study are available from the corresponding author on reasonable request.

## Abbreviations

MSU: Monosodium urate
BMDMs: Bone marrow-derived macrophages
DHE: Dihydroethidium
Drp1: Dynamin-related protein 1
ELISA: Enzyme-linked immunosorbent assay
LPS: Lipopolysaccharide
MTG: MitoTracker-Green
NLPR3: NLR family, pyrin domain-containing
OCR: Oxygen consumption rate
ROS: Reactive oxygen species
TNF-a: Tumor necrosis factor alpha
*Δψ*m: Mitochondrial membrane potential
FCM: Flow cytometry
LCM: Laser confocal microscopy
DSS: Disuccinimidyl suberate
MPO: Myeloperoxidase
SOD1: Superoxide dismutase-1
SOD-2: Superoxide dismutase-2
GPX1: Glutathione peroxidase 1
CAT: Catalase.

## Supplementary Materials

Supplementary 1 Table 1: SiRNA sequences targeting Drp1, Pink1, and Becn1. Details of the antibodies used in the western blotting and immunofluorescence. Western blotting was performed using the following antibodies: anti-LC3A/B(D3U4C, CST), anti-P62(382862, ZENBIO, Chengdu, China), anti-PINK1 (ER1706-27, HUABIO, Hangzhou, China), anti-PARKIN (ET1702-60, HUABIO, Hangzhou, China), anti-OPTN (ER2001-02, HUABIO, Hangzhou, China), anti-Beclin1 (AP0768, Bioworld), anti-p-DRP1 (3455 s, CST), anti-DRP1 (Bs7390, Bioworld), anti-GPX1 (ET1701-84, HUABIO, Hangzhou, China), anti-CAT (ER40125, HUABIO, Hangzhou, China), anti-SOD1(ER1706-49, HUABIO, Hangzhou, China), anti-SOD2 (501340, ZENBIO, Chengdu, China), anti-NLRP3 (ET1610-93, HUABIO, Hangzhou, China), anti-caspase-1 (ET1608-69, HUABIO, Hangzhou, China), anti-IL-1*β* (Asp117, CST), anti-ASC (AG-37B-0001, AdipoGen), anti-FIS1 (10956-1-AP, Proteintech), anti-OPA1 (ET1705-9, HUABIO, Hangzhou, China), anti-MFN1 (ET1702-01, HUABIO, Hangzhou, China), anti-MFN2 (ER1802-23, HUABIO, Hangzhou, China), anti-VDAC1 (ET1601-20, HUABIO, Hangzhou, China), anti-GAPDH (ET1601-4, HUABIO, Hangzhou, China), and anti-*α*-tubulin (ET1602-4, HUABIO, Hangzhou, China). The following antibodies are used for immunofluorescence: anti-ASC (sc-514414011, Santa Cruz Biotechnology), anti-LC3 (ET1609-26, HUABIO, Hangzhou, China), anti-CD11b (BM3925, BosterBio, Wuhan, China), and anti-MPO (208670, Abcam).

Supplementary 2 Supplementary Figure 1: the relationship among mitochondrial damage, mitochondrial fission, and mitophagy.

Supplementary 3 Supplementary Figure 2: Drp1 overexpression aggravates MSU crystal-mediated mitochondrial stress. (a, b) BMDMs were transfected with pcDNA3.1 plasmid or pcDNA3.1plasmid containing Drp1 open reading frame for 48 h, primed with LPS (100 ng/ml, 1 h), and then challenged with MSU suspension (75 *μ*g/ml) for 12 h. (a) Oxygen consumption rate was measured using a Seahorse XFe24 Analyzer. (b) Bar graph showing individual mitochondrial function parameters calculated from data in the panel. ^∗^*P* < 0.05 vs. without MSU crystals treatment; ^#^*P* < 0.05 vs. MSU crystals treatment + vehicle.

## References

[1] Rees F., Hui M., Doherty M. (2014). Optimizing current treatment of gout. *Nature Reviews Rheumatology*.

[2] Kuo C. F., Grainge M. J., Mallen C., Zhang W., Doherty M. (2015). Rising burden of gout in the Uk but continuing suboptimal management: a nationwide population Study. *Annals of the Rheumatic Diseases*.

[3] Chen-Xu M., Yokose C., Rai S. K., Pillinger M. H., Choi H. K. (2019). Contemporary prevalence of gout and hyperuricemia in the united states and decadal trends: the National Health and Nutrition Examination Survey, 2007-2016. *Arthritis & Rheumatology*.

[4] Liu R., Han C., Wu D. (2015). Prevalence of hyperuricemia and gout in mainland china from 2000 to 2014: a systematic review and meta-analysis. *BioMed Research International*.

[5] Richette P., Doherty M., Pascual E. (2017). 2016 updated eular evidence-based recommendations for the management of gout. *Annals of the Rheumatic Diseases*.

[6] Dalbeth N., Choi H. K., Joosten L. A. B. (2019). Gout. *Nature Reviews: Disease Primers*.

[7] Chai W., Tai Y., Shao X. (2018). Electroacupuncture alleviates pain responses and inflammation in a rat model of acute gout arthritis. *Evidence-Based Complementary and Alternative Medicine*.

[8] Terkeltaub R. (2010). Update on gout: new therapeutic strategies and options. *Nature Reviews Rheumatology*.

[9] Yin C., Liu B., Wang P. (2020). Eucalyptol alleviates inflammation and pain responses in a mouse model of gout *arthritis*. *Journal de Pharmacologie*.

[10] Trevisan G., Hoffmeister C., Rossato M. F. (2014). Trpa1 receptor stimulation by hydrogen peroxide is critical to trigger hyperalgesia and inflammation in a model of acute gout. *Free Radical Biology and Medicine*.

[11] Berman S. B., Pineda F. J., Hardwick J. M. (2008). Mitochondrial fission and fusion dynamics: the long and short of it. *Cell Death & Differentiation*.

[12] Youle R. J., van der Bliek A. M. (2012). Mitochondrial fission, fusion, and stress. *Science*.

[13] Fan X., Hussien R., Brooks G. A. (2010). H_2_o_2_-Induced mitochondrial fragmentation in C_2_c_12_ myocytes. *Free Radical Biology and Medicine*.

[14] Wu S., Zhou F., Zhang Z., Xing D. (2011). Mitochondrial oxidative stress causes mitochondrial fragmentation via differential modulation of mitochondrial fission-fusion proteins. *The FEBS Journal*.

[15] Jendrach M., Mai S., Pohl S., Vöth M., Bereiter-Hahn J. (2008). Short- and long-term alterations of mitochondrial morphology, dynamics and Mtdna after transient oxidative Stress. *Mitochondrion*.

[16] Xu S., Wang P., Zhang H. (2016). Camkii induces permeability transition through Drp1 phosphorylation during chronic beta-Ar stimulation. *Nature Communications*.

[17] Lemasters J. J. (2005). Selective mitochondrial autophagy, or mitophagy, as a targeted defense against oxidative stress, mitochondrial dysfunction, and aging. *Rejuvenation Research*.

[18] Pickles S., Vigie P., Youle R. J. (2018). Mitophagy and quality control mechanisms in mitochondrial maintenance. *Current Biology*.

[19] Wei H., Liu L., Chen Q. (2015). Selective removal of mitochondria via mitophagy: distinct pathways for different mitochondrial stresses. *Biochimica et Biophysica Acta (BBA)-Molecular Cell Research*.

[20] Yoshii S. R., Mizushima N. (2015). Autophagy machinery in the context of mammalian mitophagy. *Biochimica Et Biophysica Acta (BBA)-Molecular Cell Research*.

[21] Wong Y. C., Holzbaur E. L. (2015). Temporal dynamics of Park2/Parkin and Optn/Optineurin recruitment during the mitophagy of damaged mitochondria. *Autophagy*.

[22] Yamashita S. I., Jin X., Furukawa K. (2016). Mitochondrial division occurs concurrently with Autophagosome formation but independently of Drp1 during mitophagy. *Journal of Cell Biology*.

[23] Zhang X., Goncalves R., Mosser D. M. (2008). The isolation and characterization of murine macrophages. *Current Protocols in Immunology*.

[24] He Y., Zeng M. Y., Yang D., Motro B., Núñez G. (2016). Nek7 Is an essential mediator of Nlrp3 activation downstream of potassium efflux. *Nature*.

[25] Thangaraj A., Periyasamy P., Guo M. L., Chivero E. T., Callen S., Buch S. (2020). Mitigation of cocaine-mediated mitochondrial damage, defective mitophagy and microglial activation by superoxide dismutase mimetics. *Autophagy*.

[26] Chen B., Li H., Ou G., Ren L., Yang X., Zeng M. (2019). Curcumin attenuates Msu crystal-induced inflammation By Inhibiting The degradation Of IkappaB alpha and blocking mitochondrial Damage. *Arthritis Research & Therapy*.

[27] Kashatus D. F., Lim K. H., Brady D. C., Pershing N. L. K., Cox A. D., Counter C. M. (2011). Rala and Ralbp1 regulate mitochondrial fission at mitosis. *Nature Cell Biology*.

[28] Rong R., Yang R., Li H. (2022). The roles of mitochondrial dynamics and Nlrp3 inflammasomes in the pathogenesis of retinal light damage. *Annals of the New York Academy of Sciences*.

[29] Wu K. K. L., Long K., Lin H. (2021). The Appl1-Rab5 axis restricts Nlrp3 inflammasome activation through early endosomal-dependent mitophagy in macrophages. *Nature Communications*.

[30] Fernandes-Alnemri T., Wu J., Yu J. W. (2007). The pyroptosome: a supramolecular assembly of asc dimers mediating inflammatory cell death Via caspase-1 activation. *Cell Death & Differentiation*.

[31] Srinivasula S. M., Poyet J. L., Razmara M., Datta P., Zhang Z. J., Alnemri E. S. (2002). The PYRIN-CARD Protein ASC Is an Activating Adaptor for Caspase-1. *Journal of Biological Chemistry*.

[32] Hu J., Zhang Y., Jiang X. (2019). Ros-mediated activation and mitochondrial translocation of Camkii contributes to Drp1-dependent mitochondrial fission and apoptosis in triple-negative breast cancer cells by isorhamnetin and chloroquine. *Journal of Experimental & Clinical Cancer Research*.

[33] Shi L., Ji Y., Zhao S. (2021). Crosstalk between reactive oxygen species and dynamin-related protein 1 in periodontitis. *Free Radical Biology and Medicine*.

[34] Li J., Chang X., Shang M. (2021). The crosstalk between Drp1-dependent mitochondrial fission and oxidative stress triggers hepatocyte apoptosis induced by silver nanoparticles. *Nanoscale*.

[35] Canto C. (2018). Mitochondrial dynamics: shaping metabolic adaptation. *Cellular and Molecular Biology*.

[36] Zhang Z., Liu L., Jiang X., Zhai S. D., Xing D. (2016). The essential role of Drp1 and its regulation by S-nitrosylation of parkin in dopaminergic neurodegeneration: implications for Parkinson's disease. *Antioxidants & Redox Signaling*.

[37] Almeida A. S., Vieira H. L. A. (2017). Role of cell metabolism and mitochondrial function during adult neurogenesis. *Neurochemical Research*.

[38] Youle R. J., Narendra D. P. (2011). Mechanisms of mitophagy. *Nature Reviews Molecular Cell Biology*.

[39] Jang J. Y., Blum A., Liu J., Finkel T. (2018). The role of mitochondria in aging. *The Journal of Clinical Investigation*.

[40] Bonello F., Hassoun S. M., Mouton-Liger F. (2019). Lrrk2 impairs Pink1/parkin-dependent mitophagy via its kinase activity: pathologic insights into Parkinson's disease. *Human Molecular Genetics*.

[41] Niu K., Fang H., Chen Z. (2020). Usp33 deubiquitinates Prkn/Parkin and antagonizes its role in mitophagy. *Autophagy*.

[42] Padman B. S., Nguyen T. N., Uoselis L., Skulsuppaisarn M., Nguyen L. K., Lazarou M. (2019). Lc3/Gabaraps drive ubiquitin-independent recruitment of optineurin and Ndp52 to amplify mitophagy. *Nature Communications*.

[43] McWherter C., Choi Y. J., Serrano R. L., Mahata S. K., Terkeltaub R., Liu-Bryan R. (2018). Arhalofenate acid inhibits monosodium urate crystal-induced inflammatory responses through activation of Amp-activated protein kinase (Ampk) Signaling. *Arthritis Research & Therapy*.

[44] Crisan T. O., Cleophas M. C. P., Novakovic B. (2017). Uric acid priming in human monocytes is driven by the Akt-Pras40 autophagy pathway. *Proceedings of the National Academy of Sciences of the United States of America*.

[45] Choe J. Y., Jung H. Y., Park K. Y., Kim S. K. (2014). Enhanced P62 expression through impaired proteasomal degradation is involved in caspase-1 activation in monosodium urate crystal-induced interleukin-1b expression. *Rheumatology*.

[46] Fan W., Chen S., Wu X., Zhu J., Li J. (2021). Resveratrol relieves gouty arthritis by promoting mitophagy to inhibit activation of Nlrp3 inflammasomes. *Inflammation Research*.

[47] Cai X., Liu Y., Hu Y. (2018). ROS-mediated lysosomal membrane permeabilization is involved in bupivacaine- induced death of rabbit intervertebral disc cells. *Redox Biology*.

[48] Kleele T., Rey T., Winter J. (2021). Distinct fission signatures predict mitochondrial degradation or biogenesis. *Nature*.

[49] Li W., Feng J., Gao C. (2019). Nitration of Drp1 provokes mitophagy activation mediating neuronal injury in experimental autoimmune encephalomyelitis. *Free Radical Biology and Medicine*.

[50] Sekine S., Youle R. J. (2018). Pink1 import regulation; a fine system to convey mitochondrial stress to the cytosol. *BMC Biology*.

[51] Chen G., Han Z., Feng D. (2014). A regulatory signaling loop comprising the Pgam5 Phosphatase and Ck2 controls receptor-mediated mitophagy. *Molecular Cell*.

[52] Zhong Z., Umemura A., Sanchez-Lopez E. (2016). Nf-Kappab restricts inflammasome activation via elimination of damaged mitochondria. *Cell*.

[53] Saitoh T., Akira S. (2016). Regulation of inflammasomes by *autophagy*. *Clinical Immunology*.

[54] Ko M. S., Yun J. Y., Baek I. J. (2021). Mitophagy deficiency increases Nlrp3 to induce brown fat dysfunction in mice. *Autophagy*.

[55] Mastrocola R., Penna C., Tullio F. (2016). Pharmacological inhibition of NLRP3 inflammasome attenuates myocardial ischemia/reperfusion injury by activation of RISK and mitochondrial pathways. *Oxidative Medicine and Cellular Longevity*.

[56] Penna R. C., Aragno M., Cento A. S. (2020). Ticagrelor conditioning effects are not additive to cardioprotection induced by direct NLRP3 inflammasome inhibition: role of RISK, NLRP3, and redox cascades. *Oxidative Medicine and Cellular Longevity*.

