# Supplementary material for: Dynamin-Related Protein 1 Is Involved in Mitochondrial Damage, Defective Mitophagy, and NLRP3 Inflammasome Activation Induced by MSU Crystals
Source: Oxid Med Cell Longev. 2022 Oct 25;2022:5064494. doi: 10.1155/2022/5064494 (PMC9627272; doi:10.1155/2022/5064494)
Supplement: Supplementary 1 — Table 1: SiRNA sequences targeting Drp1, Pink1, and Becn1. Details of the antibodies used in the western blotting and immunofluorescence. Western blotting was performed using the following antibodies: anti-LC3A/B(D3U4C, CST), anti-P62(382862, ZENBIO, Chengdu, China), anti-PINK1 (ER1706-27, HUABIO, Hangzhou, China), anti-PARKIN (ET1702-60, HUABIO, Hangzhou, China), anti-OPTN (ER2001-02, HUABIO, Hangzhou, China), anti-Beclin1 (AP0768, Bioworld), anti-p-DRP1 (3455 s, CST), anti-DRP1 (Bs7390, Bioworld), anti-GPX1 (ET1701-84, HUABIO, Hangzhou, China), anti-CAT (ER40125, HUABIO, Hangzhou, China), anti-SOD1(ER1706-49, HUABIO, Hangzhou, China), anti-SOD2 (501340, ZENBIO, Chengdu, China), anti-NLRP3 (ET1610-93, HUABIO, Hangzhou, China), anti-caspase-1 (ET1608-69, HUABIO, Hangzhou, China), anti-IL-1β (Asp117, CST), anti-ASC (AG-37B-0001, AdipoGen), anti-FIS1 (10956-1-AP, Proteintech), anti-OPA1 (ET1705-9, HUABIO, Hangzhou, China), anti-MFN1 (ET1702-01, HUABIO, Hangzhou, China), anti-MFN2 (ER1802-23, HUABIO, Hangzhou, China), anti-VDAC1 (ET1601-20, HUABIO, Hangzhou, China), anti-GAPDH (ET1601-4, HUABIO, Hangzhou, China), and anti-α-tubulin (ET1602-4, HUABIO, Hangzhou, China). The following antibodies are used for immunofluorescence: anti-ASC (sc-514414011, Santa Cruz Biotechnology), anti-LC3 (ET1609-26, HUABIO, Hangzhou, China), anti-CD11b (BM3925, BosterBio, Wuhan, China), and anti-MPO (208670, Abcam). [file 5064494.f1.docx]

**Table 1 SiRNA sequences targeting Drp1, Pink1 and Becn1**

| **Gene** | **Forward sequence (5′–3′)** | **Reverse sequence (5′–3′)** |
| --- | --- | --- |
| Mouse Drp1 siRNA | AACGCAGAGCAGCGGAAAGAGTT | UUG AGAAAAAGCGAAGAGCCATT |
| Mouse Pink1 siRNA | GCGGUAAUUGACUACAGCAAATT | UUUGCUGUAGUCAAUUACCGCTT |
| Mouse Becn1 siRNA | CGGACAGUUUGGCACAAUCAATT | UUGAUUGUGCCAAACUGUCCGTT |
