# Supplementary material for: Dynamin-Related Protein 1 Is Involved in Mitochondrial Damage, Defective Mitophagy, and NLRP3 Inflammasome Activation Induced by MSU Crystals
Source: Oxid Med Cell Longev. 2022 Oct 25;2022:5064494. doi: 10.1155/2022/5064494 (PMC9627272; doi:10.1155/2022/5064494)
Supplement: Supplementary 2 — Supplementary Figure 1: the relationship among mitochondrial damage, mitochondrial fission, and mitophagy. [file 5064494.f2.pdf]

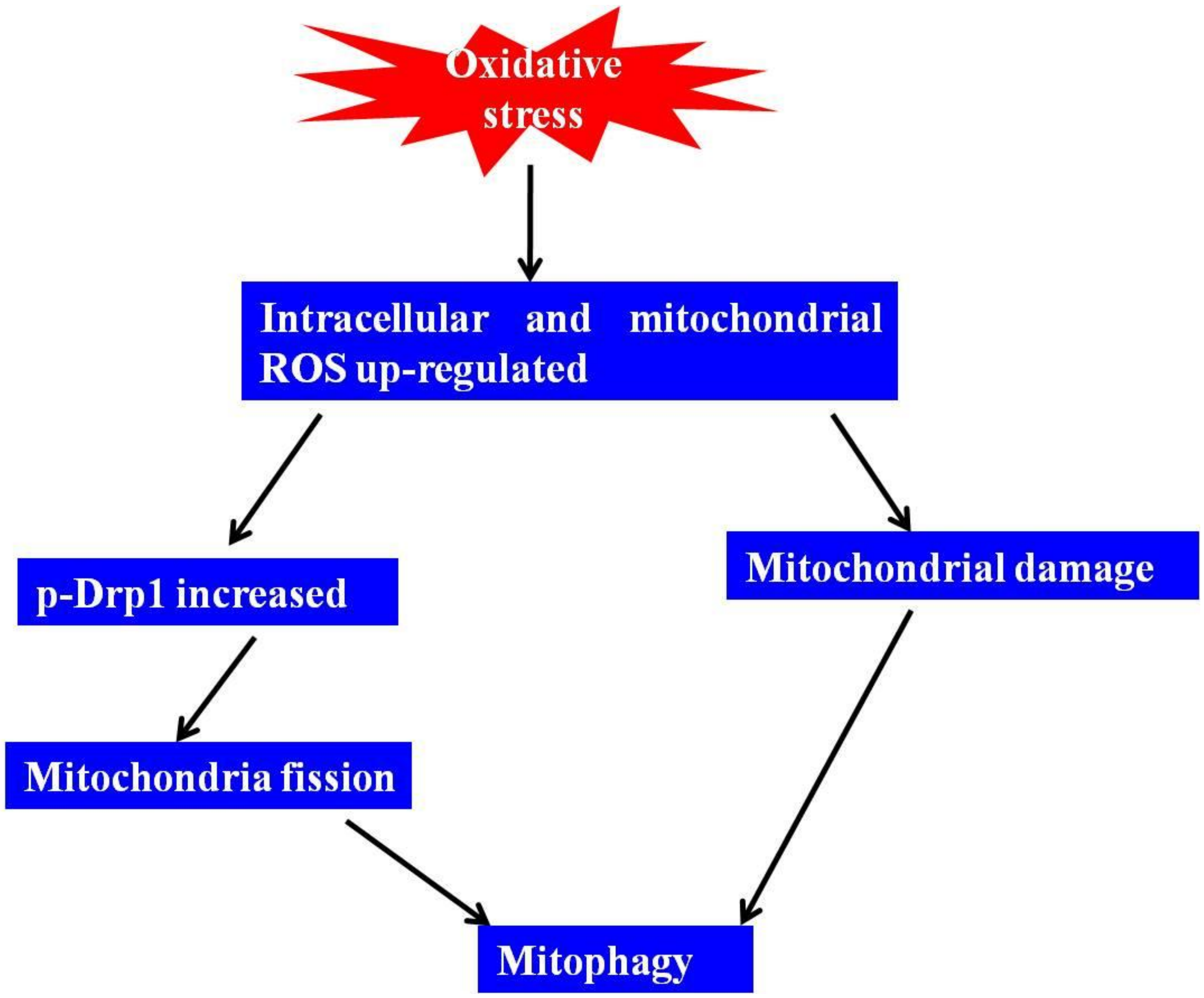
Oxidative stress
Intracellular and mitochondrial ROS up-regulated
p-Drp1 increased
Mitochondrial damage
Mitochondria fission
Mitophagy
