# Supplementary figures and images for: Dynamin-Related Protein 1 Is Involved in Mitochondrial Damage, Defective Mitophagy, and NLRP3 Inflammasome Activation Induced by MSU Crystals
Source: Oxid Med Cell Longev. 2022 Oct 25;2022:5064494. doi: 10.1155/2022/5064494 (PMC9627272; doi:10.1155/2022/5064494)

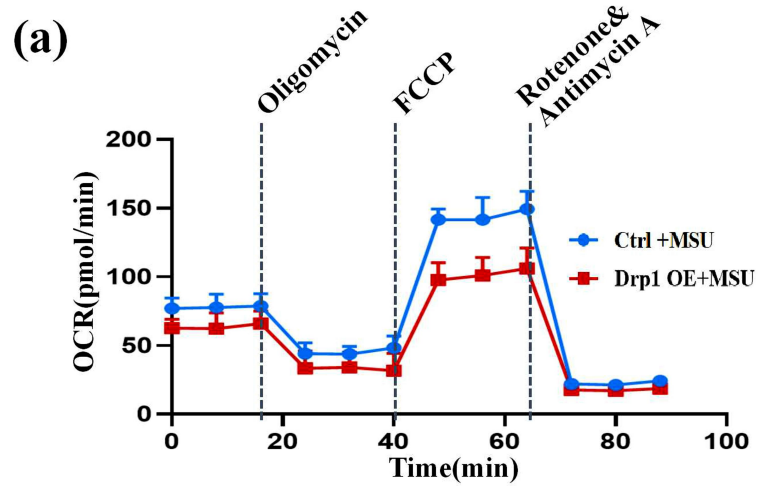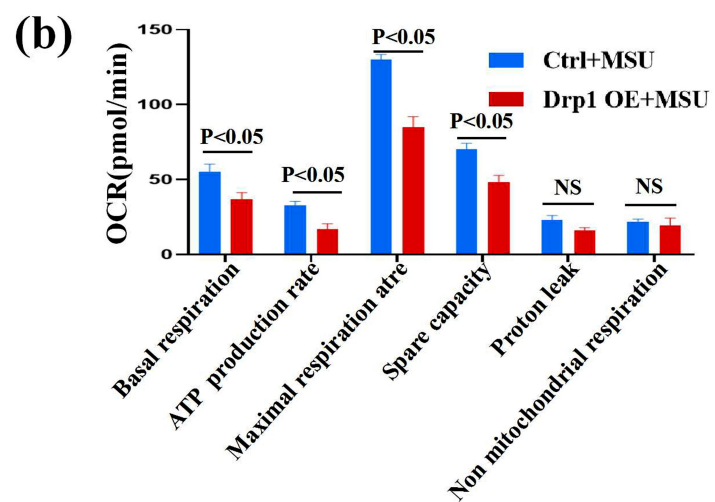

Supplement: Supplementary 3 — Supplementary Figure 2: Drp1 overexpression aggravates MSU crystal-mediated mitochondrial stress. (a, b) BMDMs were transfected with pcDNA3.1 plasmid or pcDNA3.1plasmid containing Drp1 open reading frame for 48 h, primed with LPS (100 ng/ml, 1 h), and then challenged with MSU suspension (75 μg/ml) for 12 h. (a) Oxygen consumption rate was measured using a Seahorse XFe24 Analyzer. (b) Bar graph showing individual mitochondrial function parameters calculated from data in the panel. ∗P < 0.05 vs. without MSU crystals treatment; #P < 0.05 vs. MSU crystals treatment + vehicle. [file 5064494.f3.pdf]
